# Developing and testing measures of reproductive decision-making agency in Nepal

**DOI:** 10.1016/j.ssmph.2019.100473

**Published:** 2019-11-20

**Authors:** Laura Hinson, Jeffrey Edmeades, Lydia Murithi, Mahesh Puri

**Affiliations:** a Social and Behavioral Scientist, International Center for Research on Women, 1120 20th St NW, Washington, DC, 20036, USA; b Independent Consultant, USA; c Pathfinder International, Pathfinder International & Evidence to Action Project, 1250 23rd Street NW, Suite 475, Washington, DC, 20037, USA; d Center for Research on Environment, Health and Population Activities, P.O.Box. 9626, Kusunti (near Yatayat Office), Lalitpur, Nepal

## Abstract

Conceptual ambiguity in how we define reproductive empowerment has left the field with inconclusive evidence of its relationship to key reproductive outcomes. Our study aimed to develop and test a measure of reproductive decision-making agency, which is a critical component of reproductive empowerment, in a sample of married women from two Nepalese districts. Initial measures were developed based on theory and previous literature. Next, we used cognitive interviewing techniques to explore local meanings of reproductive empowerment and decision making through eight focus group discussions and 24 in-depth interviews. This process resulted in four core questions used to assess decision making across three domains of reproductive behavior: when to have children, whether to use family planning, and which family planning method to use. We combined these questions to develop an overall assessment of decision-making agency. These measures were included in a quantitative survey conducted with 1000 women, split evenly between the two districts. The combined measure of overall reproductive decision-making agency was internally consistent across the three domains examined (Cronbach's alpha = 0.6416), performed well across a range of validity assessments, including those aimed at assessing construct and criterion validity, and was predictive of a range of reproductive outcomes, particularly those related to feelings of reproductive control. The results suggest that the measures developed here provide insight into the nuances of joint versus sole decision making beyond those provided by standard measures. With better measures of reproductive agency, we can better design interventions for men and women, to meet their reproductive needs.

## Highlights

• New measures of reproductive decision-making agency developed using data from Nepal.
• New measures are predictive of reproductive self-efficacy and feelings of control.
• Comparison with other measures suggest ambiguity of meaning of joint-decisions.
• Measures offer insights into empowerment beyond what standard measures show.

## Background

In recent decades, the development field has increasingly recognized the role that women's empowerment plays in shaping reproductive outcomes, particularly in areas of the world where women are more disempowered than men. Although several studies have found a positive association between increased empowerment and a range of reproductive outcomes—including lower fertility, longer birth intervals, use of contraception, and lower rates of unintended pregnancy (e.g. Upadhyay & Hindin, 2005)—the overall empirical evidence for this association is more mixed than the theoretical consensus would suggest (Upadhyay, Dworkin, Weitz, & Foster, 2014; James-Hawkins, Peters, VanderEnd, Bardin, & Yount, 2016; Pratley, 2016). To a significant extent, this reflects an ambiguity regarding how empowerment is defined, measured, and operationalized in the reproductive sphere (Malhotra & Schuler, 2005; Upadhyay et al., 2014; Pratley, 2016). For example, it is unclear whether authors using the terms “reproductive autonomy,” “women's agency,” “reproductive rights,” or “reproductive control” are referring to the same or related concepts, particularly as these are often measured differently. As a result, researchers, policy makers, and health practitioners have struggled to fully understand the conditions under which women's empowerment shapes specific reproductive outcomes, limiting their ability to develop effective interventions.

Edmeades, Mejia, and Sebany (2018) propose a conceptual framework for reproductive empowerment that address some of these challenges through positioning reproductive empowerment as a distinct dimension of overall empowerment, building on, among others, conceptual frameworks of women's empowerment (see, for example, van Eerdewijk et al., 2017; Kabeer, 2001). Within this approach, reproductive empowerment results from the interaction of three interrelated, multilevel processes: *voice*, the capacity of individuals to assert their interests, articulate their opinions and desires, and meaningfully participate in decision-making processes related to their reproductive lives; *choice*, the ability of individuals to meaningfully contribute to reproductive decisions; and *power*, the ability of individuals to shape reproductive decision-making processes by exerting influence over others, which acts as a key enabler of both voice and choice. Three key expressions of empowerment are particularly relevant in the reproductive sphere: *collective action*, the ability of groups to shape reproductive policy and practice through group advocacy; *leadership*, the degree to which individuals and groups play a lead role in debates about reproduction; and *decision-making*, the ability of individuals to meaningfully engage in the decision-making process.

Of these three expressions of empowerment, decision making has received the most attention from researchers focused on reproductive outcomes, with most literature exploring the influence of women's engagement in, and control over, specific decisions on a range of reproductive outcomes. As is the case for empowerment more generally, the evidence for the effect of decision making on reproductive outcomes is more mixed than the theoretical consensus would suggest (Upadhyay et al., 2014; Pratley, 2016). This inconsistency reflects a lack of consensus in the field about which aspects of the decision-making process are most reflective of empowerment and how to measure agency and empowerment within the context of reproductive processes.

Much of the evidence on decision making has focused broadly on decisions related to household functions (e.g., from the Demographic and Health Surveys [DHS]) rather than those specific to reproduction, implicitly assuming reproductive decisions follow similar processes (Malhotra & Schuler, 2005). When focused more specifically on reproduction, decision making questions have tended to rely on a single question aimed at understanding who typically makes the final decision on a specific topic. An example of this is the cross-national Performance Monitoring and Accountability 2020 (PMA2020) questionnaires, which ask specifically about decisions related to contraceptive use.

Although the single-question approach allows for direct measurement of decision making rather than relying on proxies, it remains unclear how these measures are related to the broader concepts of agency and empowerment or how to interpret the responses, which often are dependent on the judgment of individual researchers. This is particularly the case when responses are divided into the usual categories (mainly husband, mainly participant, or joint). Often it is unclear whether sole or joint decision making represents greater agency for any given decision. For example, women who make reproductive decisions alone may include women with high agency along with those forced to make decisions alone because of a lack of broader agency (as could be argued for covert use of contraception, for example). Alternatively, women reporting joint decision making may be only peripherally involved in the decision because of power imbalances in their relationship or fully engaged as equal partners. In the absence of additional information on the decision-making process, the researcher is forced to make assumptions about which responses represent empowerment or to adopt simplified measures of decision making, both of which are problematic for accurate measurement.

Finally, most research has focused on linking decision-making agency to outcomes assumed to be responsive to changes in women's agency through indirect causal pathways, such as current use of modern contraception, rather than outcomes more directly linked to the process of decision-making. Edmeades et al. (2018) suggest that more appropriate outcomes are those that explicitly seek to understand how individuals want to be involved in decisions and how closely the outcomes match their reproductive desires, hewing closely to the roles of voice, power, and choice in understanding empowerment. When viewed from this perspective, the choice *not* to use a contraceptive method may be as reflective of agency as a decision *to* use one. As a result, some of the inconsistency in the predictive ability of measures of agency may reflect erroneous assumptions about the relationship between decision-making agency and specific reproductive behaviors or outcomes.

In this study, we addressed these issues by developing and testing measures that capture women's decision-making agency across multiple domains of reproductive health. We explicitly aimed to capture core components of empowerment in the decision-making process by including elements of voice, power, and choice in our measures (Edmeades et al., 2018). We used these measures to examine the relationship of empowerment in decision making to key reproductive outcomes, to determine the advantages our measures have compared with standard measures, and to illuminate the meaning behind joint versus sole decision making for women.

## Methods

The data for this mixed-method study come from the Morang and Kaski Districts in Nepal, which were purposively selected to obtain a diverse sample on which to develop and refine measures of reproductive decision making and to capture the significant variation in the cultural, economic, social, and migration contexts within which reproductive decisions are made in Nepal. Morang is in Province 1 in Nepal's lowland area, while Kaski is in Province 4 in Nepal's hill areas. Both are predominantly rural districts with relatively large urban centers and large numbers of migrants from surrounding areas. Both provinces have low fertility rates (2.0 and 2.3 for Provinces 1 and 4, respectively) and relatively high rates of modern contraceptive use (55% and 49% of women aged 15–49 years in Province 1 and 4, respectively), compared with the national average of 43%. Around one-quarter of women have an unmet need for contraception. In both provinces, according to the DHS, around two-thirds of women reported that their contraceptive decisions were made jointly with their husbands, although the percentage was slightly higher in Province 1 (Ministry of Health, 2017).

Participants for the qualitative and quantitative samples were drawn from the same areas to ensure comparability, although no individuals were included in both samples. The qualitative sample included men (aged 18–59 years) and women (aged 18–45 years). The quantitative sample included women aged 20–35 years who had been married for at least six months and who currently lived with their partners. We restricted age to capture people who were likely to be actively engaged in a range of decisions about childbearing and contraceptive use. Participants for focus group discussions (FGDs) and in-depth interviews (IDIs) were purposively selected in consultation with community leaders.

For both the qualitative and the quantitative research activities, site selection and sampling were based on a four-stage process. In the first stage, one municipality was purposively selected from each district based on their sociodemographic and economic characteristics. In the second stage, 20 wards were purposively selected on the same basis as the municipality. Individuals for the qualitative sample were recruited within two of these wards. These same wards were divided into between three and ten segments, depending on number of households in each segment. Finally, for the quantitative sample, participants were selected randomly within each segment, with 25 individuals interviewed in each segment. We screened 2782 households to find 1000 eligible women to participate.

In total, we conducted eight FGDs with 64 participants, 20 IDIs, evenly split between men and women, and 1000 quantitative surveys with women^1^, all equally split between the two sites. We conducted this work between June and August 2017. Thirteen women refused to participate in the quantitative survey and were replaced to reach the total sample.

Ethical approval was obtained from the International Center for Research on Women Internal Review Board (IRB) and the Nepal Health Research Council.

### Study design

We conducted this study in two phases. In the first phase, we drafted set of quantitative measures of reproductive decision making and then refined them using insights drawn from our qualitative sample. In the second phase, we assessed the internal consistency and validity of these measures through a series of exploratory statistical analyses, using our quantitative survey data.

### Phase 1: developing and refining the quantitative reproductive decision-making measures

We sought to develop measures that would adequately capture the degree to which individuals are *meaningfully* engaged in the decision-making process, and their level of satisfaction with their own influence over the decision itself, building on the reproductive empowerment framework developed by Edmeades et al. (2018). To do so, we built on several existing approaches to decision making in areas other than reproductive behavior, such as the Women's Empowerment in Agriculture Index (WEAI), as well as the measures used in the DHS and other questionnaires that examine reproductive autonomy or decision making (e.g. Upadhyay et al., 2014) to expand on standard approaches used in the field. This approach was reviewed by a multidisciplinary group of experts who also made suggestions for how to approach examining decision making within the context of the framework.

Because we wanted our measures to capture a range of commonly made reproductive decisions, we centered the decision-making process on five domains of reproductive behavior. These domains were developed based on the literature on women's empowerment and on family planning (see Edmeades et al., 2018). In this analysis, we focus on three domains of reproductive decision making that the qualitative research and preliminary analyses of the quantitative data found were of particular relevance to the married women in our sample: when to have children, whether to use family planning, and which method of family planning to use.

Eleven questions were initially developed for each domain, collectively designed to capture key components of the decision-making process from start to finish. These questions, which we refer to as a “question set,” were designed to elicit information on the topic of the discussion (e.g., when to have children), who was involved in the discussion, which individuals had an influence on the decision, whether there was a difference in opinion between the woman and her spouse, who made the final decision, and the outcome of the decision (e.g. had a child or did not). The goal of the question set was to collect information on voice, power, and choice—along with other aspects—within the process of making decisions.

To assess how these questions directly related to core elements of the conceptual framework and to examine how the question sets were interpreted in the Nepalese context prior to applying them in a quantitative survey, we collected qualitative data from similar groups to those we wanted to include in the quantitative component of the study. We first elicited information through FGDs on general perceptions of empowerment within the reproductive sphere, what the “typical” decision-making process for each domain involved, and what was viewed as an optimal decision-making process. We used findings from these discussions to inform the IDIs, during which we utilized cognitive interviewing techniques to explore in greater depth how participants interpreted the nuances of the questions. For each question set administered during the IDI, participants were asked open-ended questions to explore the cognitive process they engaged in when answering questions, including their understanding of specific meanings of terms/words and key concepts and how they recalled past events.

This analysis resulted in several changes to the wording and structure of the question set for each domain, as well as the addition of questions to the set. Based on the qualitative analyses and theory, we identified four core questions as best capturing key components of the decision-making process, particularly in terms of voice, power, and choice: whether the participant shared her opinion on the decision (and, if not, why not) with others; whether the participant felt her opinion was considered when the decision was made; who made the final decision; and whether the participant was satisfied with the decision-making process (See Appendix 1 for these questions for Domain 1: When to Have Children).

Finally, the revised question set was applied to each domain and incorporated into a quantitative survey that included questions on demographic, relationship, contraceptive, and fertility characteristics, among other topics.

### Phase 2: testing and validating reproductive decision-making agency measures

We used several methods for testing and validating measures. Once data were collected and cleaned, we conducted internal consistency and validity tests in stages, beginning with assessing the internal consistency of the question set across and within domains, with other variables in the data set, against the men's data, and compared with our qualitative findings. Based on these findings, we created measures of reproductive decision-making agency for each domain and for all the domains in combination. Next we explored to what extent our measures were associated with key reproductive outcomes related to contraceptive use and feelings of reproductive control. Finally, we sought to understand what advantages and disadvantages our measures had over existing measures. Each of these stages is described in greater detail below, and a summary of reliability and validity checks is found in Table 1.

**Table 1:** Assessment of reliability and validity of reproductive decision-making measures.

Type of assessment	Description of process and key findings
Reliability	
Internal consistency	We assessed internal consistency in two ways. First, we assessed the degree to which the reproductive decision-making measures for each of the three domains were related to each other by conducting a Cronbach's alpha test (α = 0.6416), which suggested an acceptable level of internal consistency. Second, we directly compared the results from the quantitative survey and data analyses with the patterns observed in the qualitative data collected during the cognitive interviewing phase. This comparison was done both within specific domains of reproductive decision-making agency and across the different domains. It provided strong evidence that the quantitative measures closely matched what women described as decision-making agency in the qualitative phase and individual questions and domains were interrelated in ways that were consistent with women's conceptualization of decision-making agency. Although formal tests of external reliability were not conducted in this study, the measures performed equally well in both locations tested, despite the significant social, cultural, and economic differences between them.
Validity	
Content validity	The broader conceptual framework for reproductive empowerment that provides the foundation for this measure (Edmeades et al., 2018) was reviewed at multiple points by content experts who found it to be sound and comprehensive. The identification of specific domains of reproductive behavior within which to examine decision making specifically was conducted by an interdisciplinary team of international content experts in sexual and reproductive health and has been reviewed by several experts, both prior to and following the collection of the data used in this paper.
Face validity	We assessed the face validity of the measure in several ways, including as a part of the broader assessment of content validity, by local researchers and experts in Nepal, and through multiple presentations and discussions with experts. In all cases, these reviews raised no concerns over the suitability of the measures for the purpose of better understanding reproductive decision-making agency.
Construct validity	We assessed construct validity of the measure primarily through examining the statistical relationship between the combined measure of decision-making agency and other factors that theory suggests are related to this (convergent validity). As prior research suggested, decision-making agency was found to be positively and statistically significantly associated with the woman's age (*X*^2^[4, N = 935] = 10.01; *P* = .040), her education (*X*^2^[8, N = 935] = 18.24; *P* = .020), her husband's education (*X*^2^[8, N = 935] = 21.48; *P* = .006), her employment (*X*^2^[2, N = 935] = 24.41; *P* = .000), and the number of children she had (*X*^2^[10, N = 935] = 26.11; *P* = .004). No statistically significant relationship was associated with having had sons. These relationships were confirmed through bivariate regressions in which each variable was regressed on the agency measure. Together, these assessments strongly suggested that the combined decision-making agency variable captures key aspects of the underlying construct of agency.
Criterion validity	There is no recognized “gold-standard” measure of reproductive decision making against which to assess the performance of our measure. However, there are several approaches to measuring decision making in reproductive matters that are commonly used in the field. These include those used by the Demographic and Health Surveys and the PMA2020 surveys, although neither includes questions across the full range of domains covered in our paper. In order to compare our measure to these, we developed questions for each domain that are modelled on the approach used by the PMA2020 and combined these together to create a single measure that is analogous to ours, using analytical approaches that are common in the field when using the PMA2020 decision-making variables. Although there are important differences between the two approaches in terms of how the variables themselves are constructed, statistical tests of association suggest these are related to a limited extent (*r* = 0.43; *X*^2^[4, N = 935] = 191.49; *P* = .000). This suggests that although both measures are capturing much of the same underlying construct, there are important differences that suggest additional value in the measurement approach we have developed.

### Measures

For each domain, we constructed a three-category variable indicating low, medium, or high agency based on the four core questions described above. Decisions on which combinations of responses corresponded to these levels of agency were based on the theoretical framework and insights from the qualitative data, with a priority placed on capturing meaningful engagement in the decision process, the level of satisfaction with the process and the level of direct involvement in the decision. The criteria used to categorize these levels is found in Table 2.

**Table 2:** Criteria for categorization of reproductive decision-making agency based on four core decision-making questions.

Category	Criteria
High agency	Anyone who reported that she (a) shared her opinion and felt her opinion was valued, (b) was the final decision-maker or it was joint, and (c) was satisfied (or wanted less influence) with the final decision; OR anyone who reported that she (a) did not share her opinion because she did not care about the issue or agreed already with her husband on the outcome, (b) was the final decision-maker or it was joint, and (c) was satisfied (or wanted less influence) with the final decision.
Low agency	Anyone who reported that she (a) did not share her opinion because she did not feel comfortable or did not think it would be valued or shared it but felt her opinion was not valued (or was unsure if it was valued), (b) was not involved in the final decision (i.e., it was husband or others), and (c) wanted more influence in the final decision.
Medium agency	Everyone not included in high or low agency groups.

Next, we combined the three domain-specific decision-making agency measures into a single measure by constructing an additive scale from the three domain-specific categorical variables. This resulted in a single continuous variable with values ranging from three to nine, with three indicating low agency on all three domains, and nine indicating high agency on each. We then created a three-category variable based on this continuous measure, classifying women as having high, medium, or low reproductive decision-making agency, with those scoring three or four categorized as having low agency, those scoring five, six, or seven categorized as having a medium level of agency, and those scoring eight or nine categorized as having high agency.

Within each domain, we assessed the internal consistency of the combined agency variable using a similar process to the individual questions. Overall, the combined measure correlated closely with expected outcomes and determinants of agency and showed the expected relationships across domains (results not shown).

To assess consistency of responses across items, we examined response patterns within and across domains and their relationship with other relevant factors, and by comparing the patterns of responses within each domain to those identified in the qualitative phase of the research. We assessed whether there was an intuitive, consistent pattern of responses across these different data sources. For example, that women who report their husbands were the decision-makers in one domain were likely to report that their husbands were the main decision-makers in other domains. A summary of the ways in which we assessed the reliability and validity of the measures is included in Table 1.

We explored the relationship between reproductive decision-making agency and demographic and relationship characteristics that are linked to empowerment and agency. We first assessed the statistical relationship between the decision-making agency measures and our demographic and relationship characteristics using bivariate regressions. To assess the external validity of the combined measure of agency in decision making, we looked at how our measures were correlated with two key outcomes expected to be related to the agency in reproductive decisions. The first outcome of interest was met contraceptive need, which we calculated in the same manner that is used in the DHS^2^. We chose this outcome because it is frequently used in analyses that address empowerment and women's agency and because of its close conceptual link to empowerment. The second outcome we examined was current use of modern contraception, which is often assumed to be tied to women's empowerment and has a clear potential effect on reproductive behavior. We hypothesized that greater decision-making agency would lead to both higher met need for and use of contraception.

We then explored the degree to which the measure was predictive of a range of key reproductive outcomes, focusing in particular on three different measures of feelings of reproductive control: how hopeful participants were about their ability to have control over how many children they have and when; how hopeful participants were about their ability to control fertility using a method of contraception if and when they want to; and if participants felt able to achieve their desires about when to have children up to that point in their lives, including when to stop having children. For each of these three outcomes, we hypothesized that having decision-making agency would lead to a higher belief in reaching one's reproductive desires and intentions.

Although we did examine the relationship between our measures and contraceptive use, we ultimately concentrated much of the analyses on the three outcomes above and unmet need for contraception because we feel these outcomes better reflect agency and empowerment, particularly in terms of the expression of voice and choice. We fit logistic regression models to see whether each of the domains of decision-making agency—separately and in combination—were associated with these reproductive health outcomes. All models adjusted for socioeconomic and demographic variables related to reproductive decision-making agency, including site, age, parity, education, wealth, religion, caste, woman's and husband's educations, and contraceptive use, with standard errors corrected for clustering.

Finally, we examined how each measure compared with other approaches based on more commonly used questions, using the approach taken by the PMA2020 to asking about decision making as a model. Because the PMA2020 survey does not ask questions about each of the domains we identified for reproductive empowerment, the wording and response categories used for the question on contraceptive use were adapted to each domain. In PMA2020 surveys, participants are asked who has the final decision in each of the domain topics, using the following question formulation: “Would you say that deciding [*outcome of interest*] is mainly your decision, mainly your husband/partner's decision or did you both decide together?” with the response categories of mainly participant, mainly husband, joint decision, or others. In our survey, we maintained the question and response structures but adapted the scenarios to match our domains. As there is no firm consensus in the literature about whether joint or sole decision making represents greater agency, we rely on women's expressed preference in the qualitative work for joint decision making and regard this as the highest form of agency when categorizing these questions.

To create the composite PMA2020-style measure for each of the first three domains, we followed the same process as for our own reproductive decision-making agency measure, also resulting in a continuous variable scored between three and nine. We considered women who reported that their husband or someone other than themselves mainly made decisions on the PMA-style questions to have the lowest agency, women reporting joint decision making to be the highest, and women making decisions alone representing a middle ground, following the approach most often used in the literature. From this variable, we created a categorical variable of low, medium, and high composite PMA-style decision-making agency.

To compare our composite agency measure with the PMA2020-style questions for each domain, we examined the distribution of the responses on both sets of measures, followed by a bivariate assessment of the level of association between the two variables for each domain. Next, we compared the PMA2020-style composite variable with our measure, exploring the areas of concordance and discordance between the two variables.

## Results

Our results are displayed according to the order of the analytic process described above for Phase 2. We highlight findings from the assessment of internal consistency of the measures, followed by the results of how our measures link to key reproductive outcomes and comparison of our measures to the PMA2020 measures.

Table 3 shows the distribution of responses for each of the four core items for each of three domains. Generally, levels of agency varied in the ways that we expected, based on the findings from our qualitative analyses. For each domain, most women reported that they shared their opinion about what they wanted (76.3% on the decision about when to have children, 79.5% on whether to use contraception, and 81.0% on which method to use), and the majority of those who shared their opinion felt that it was valued (78.0% for Domain 1, 82.4% Domain 2, and 78.6% for Domain 3). Women were most likely to report that their husband alone made the final decision regarding when to have children (38.2%), with contraceptive decisions more likely to be made jointly or alone. Lastly, roughly one-third of women wanted more influence in the decision in each domain (33.3%, 30.7%, and 29.9%, respectively).

**Table 3:** Four core decision-making agency questions for three reproductive health domains, among all participants who discussed that topic with their partner, Nepal, 2017

	When to Have Children (Domain 1)		Whether to Use Family Planning (Domain 2)		Which Family Planning Method to Use (Domain 3)	
	n = 991		n = 966		n = 958	
	No.	%	No.	%	No.	%
**Did you share your opinion?**						
Shared	756	76.3%	768	79.5%	776	81.0%
Didn't share: uncomfortable or didn't think would be valued	86	8.7%	72	7.5%	77	8.0%
Had same opinion as husband (or didn't care about issue)	149	15.0%	126	13.0%	105	11.0%
*Total*	*991*	*100.0%*	*966*	*100.0%*	*958*	*100.0%*
**If the participant shared their opinion:**						
**Did you think your opinion was valued?**						
Not valued or unsure	166	22.0%	135	17.6%	166	21.4%
Valued	590	78.0%	633	82.4%	610	78.6%
*Total*	*756*	*100.0%*	*768*	*100.0%*	*776*	*100.0%*
**Who had the final say?**						
Husband (or other)	374	38.2%	336	35.0%	339	35.8%
Participant	258	26.3%	348	36.3%	398	42.1%
Joint	348	35.5%	276	28.7%	209	22.1%
*Total*	*980*	*100.0%*	*960*	*100.0%*	*946*	*100.0%*

**Did you want more influence in decision?**						
No, satisfied (or wanted less)	654	66.7%	665	69.3%	663	70.1%
Yes, wanted more	326	33.3%	295	30.7%	283	29.9%
*Total*	*980*	*100.0%*	*960*	*100.0%*	*946*	*100.0%*

Note: response option in parentheses had fewer than 12 participants within a single domain and were therefore collapsed.

### Internal consistency of our agency measures

We found high levels of internal consistency within the four core questions and across the other questions in the set. For example, most women who said their mother-in-law influenced the final decision were also more likely to report that they wished they had more influence in the process. Reporting across domains was also consistent, with response patterns distributed in an intuitive way. For example, women who reported that their husband was the final decision-maker on when to have children (Domain 1) were also more likely to report that husband was final decision-maker on other domains. This was also the case when comparing the measures against key socioeconomic and demographic characteristics of the participants, such as educational attainment, age, parity, or employment status (results not shown).

Data from our question sets were also consistent with the qualitative findings. For example, qualitative findings indicated that proof of fertility, parity, and the sex of living children were the main determinants of who made decisions about when to have children and whether to use family planning, while women mostly decide which method of family planning to use (Domain 3). In addition, the data from our question sets also suggested greater complexity and ambiguity about what represents true joint decision making than would be suggested by solely looking at responses to the question on who made the final decision.

### Relationship of reproductive decision-making agency to demographic characteristics

Table 4 presents the distribution of participant characteristics by the agency measures for each domain. The general patterns of responses were consistent with *a priori* expectations. There was much higher agency among women in Kaski than among women in Morang (74.0% of women in the former region were categorized in the highest agency group vs. only 29.5% in the latter, *P* < .001). Agency increases with age (*P* = .040), with 57.5% of women aged 31–35 years being categorized as having high agency (compared with 55.5% of those aged 26–30 years and 46.7% of those aged 20–25 years; although slightly higher proportions of the older age group were also categorized as having low agency than in either of the other age groups). As expected, decision-making agency was highest among women with more education (*P* = .020), and the proportion categorized as having higher agency increased with each additional level of education; among those with a more educated husband, particularly above lower secondary level; among those with greater household wealth (*P* < .001); and among those with formal employment (vs. unemployed, *P* < .001). Women who had no children and women who had three or more children were much more likely to be in the lowest agency group (*P* = .006) than those with two children, who were most likely to be categorized as having high levels of agency.

**Table 4:** Level of reproductive decision-making agency by demographic characteristics, Nepal, 2017

		Low Agency		Medium Agency		High Agency		Total	
		n = 85		n = 362		n = 488		n = 935	
		No.	%	No.	%	No.	%	No.	%
Site***									
Morang		72	15.7%	251	54.8%	135	29.5%	458	100%
Kaski		13	2.7%	111	23.3%	353	74.0%	477	100%
Age**									
20–25 years		41	10.2%	174	43.2%	188	46.7%	403	100%
26–30 years		22	7.4%	111	37.1%	166	55.5%	299	100%
31–35 years		22	9.4%	77	33.0%	134	57.5%	233	100%
Education**									
No/informal education		14	12.5%	55	49.1%	43	38.4%	112	100%
Primary only		15	9.9%	63	41.7%	73	48.3%	151	100%
Lower secondary		36	8.5%	166	39.0%	224	52.6%	426	100%
Higher secondary		16	9.6%	55	32.9%	96	57.5%	167	100%
Bachelor's degree or above		4	5.1%	23	29.1%	52	65.8%	79	100%
Wealth tertile***									
Poorest		30	9.7%	146	47.2%	133	43.0%	309	100%
Medium		38	9.6%	143	36.3%	213	54.1%	394	100%
Richest		17	7.3%	73	31.5%	142	61.2%	232	100%
Employment***									
Not employed		70	11.2%	262	41.9%	293	46.9%	625	100%
Employed		15	4.8%	100	32.3%	195	62.9%	310	100%
Parity***									
No children		17	17.2%	38	38.4%	44	44.4%	99	100%
One child		25	7.2%	140	40.3%	182	52.4%	347	100%
Two children		25	7.1%	125	35.7%	200	57.1%	350	100%
Three or more children		18	12.9%	59	42.4%	62	44.6%	139	100%
**Has had a son**									
No son or no children		30	10.1%	123	41.3%	145	48.7%	298	100%
Has a son		55	8.6%	239	37.5%	343	53.8%	637	100%
Religion**									
Other/not Hindu		4	5.7%	19	27.1%	47	67.1%	70	100%
Hindu		81	9.4%	343	39.7%	441	51.0%	865	100%
Caste***									
Dalit	9		6.1%	44	29.9%	94	63.9%	147	100%
Janajati-hill	14		5.9%	70	29.7%	152	64.4%	236	100%
Janajati-terai	24		15.1%	91	57.2%	44	27.7%	159	100%
Madhesi/Muslim	14		15.2%	56	60.9%	22	23.9%	92	100%
Brahaman/chettri	24		8.0%	101	33.6%	176	58.5%	301	100%
Husband's education**									
No/informal education		3	5.4%	36	64.3%	17	30.4%	56	100%
Primary only		12	10.0%	51	42.5%	57	47.5%	120	100%
Lower secondary		40	9.0%	162	36.3%	244	54.7%	446	100%
Higher secondary		12	7.1%	63	37.1%	95	55.9%	170	100%
Bachelor's or above		18	12.6%	50	35.0%	75	52.4%	143	100%
Total		85	9.1%	362	38.7%	488	52.2%	935	100%

*P < .1; **P < .05; ***P < .01.

### Relationship of reproductive decision-making agency to met need for contraception and feelings of reproductive control

**Fig. 1:**
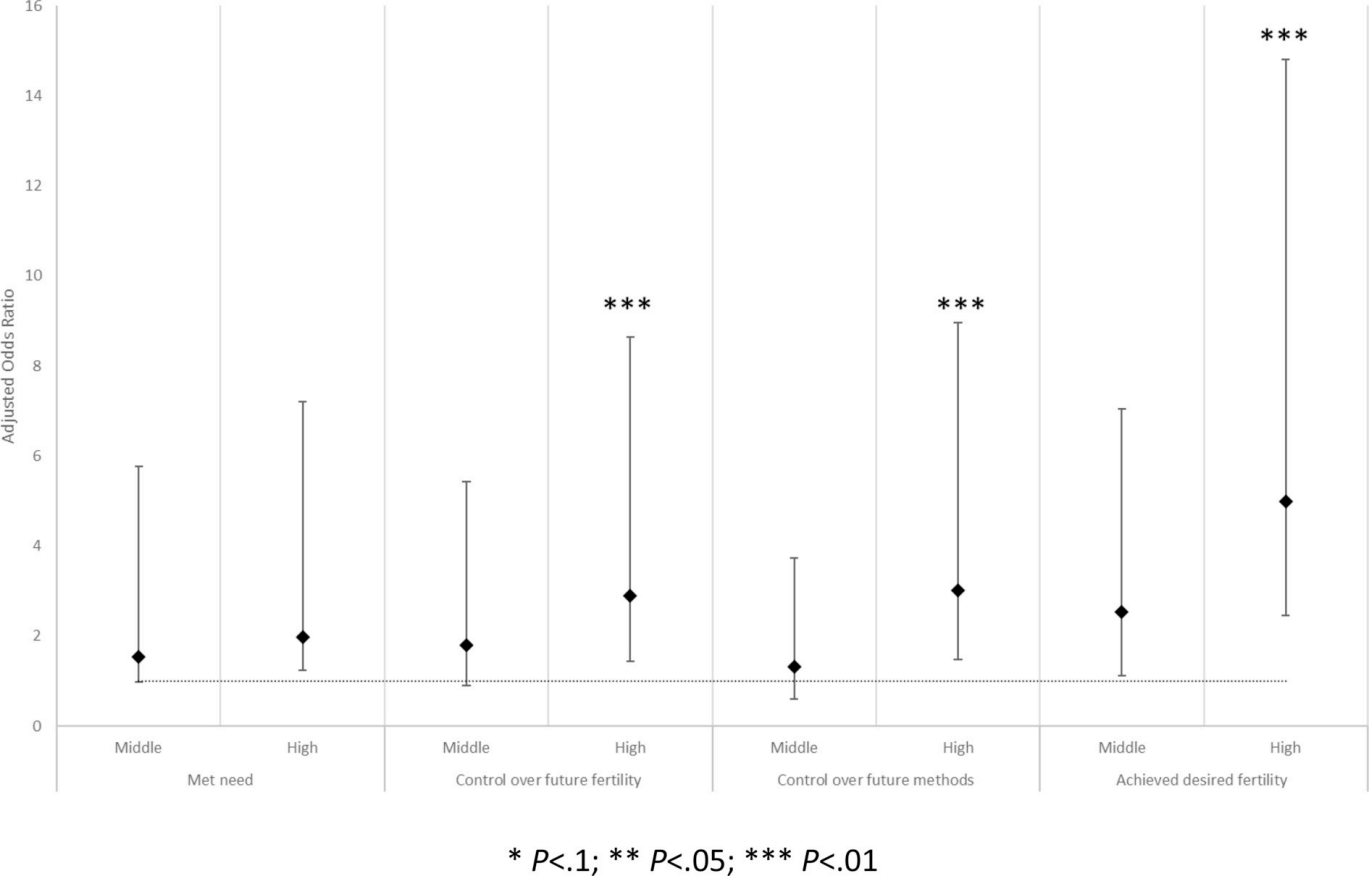
Adjusted odds ratios and 95% confidence intervals of four key outcomes by level of reproductive health decision-making agency, Nepal 2017 (reference category: Low agency) *P < .1; **P < .05; ***P < .01.

Fig. 1 presents the results of multivariate logistic regression modelling the determinants of met need for family planning and feelings of reproductive control. Women in the highest agency group were significantly more likely to have hope that they could achieve their reproductive desires, or had achieved them to date, than women in the lowest agency group. Women in the highest agency group had more than two-fold higher odds of being hopeful they could achieve their fertility desires (adjusted odds ratio [aOR] = 2.88; 95% confidence interval [CI] = 1.45–5.74; *P* = .002) and had three times higher odds of being hopeful (aOR = 3.01; 95% CI = 1.53–5.94; *P* = .001) than women with low agency. Lastly, women in the highest agency group had nearly five-fold higher odds of feeling like they had achieved their fertility desires to date (aOR = 4.98; 95% CI + 2.52–9.83; *P* < .001) than those with low agency. Although not statistically significant, the direction of the effect of the agency variable on met need for contraception was in the expected direction, with those in higher agency groups having higher odds of met need.

### Comparability with other measures of reproductive decision making

Table 5 presents the levels of concordance between the PMA2020-style composite variable with our composite measure of decision-making agency. Although there are clear areas of concordance between the two measures, as expected, there are several areas of discordance. For example, in Domain 1, only 72.1% (n = 315) of women reporting mainly joint decision making on this topic based on the PMA2020-style questions were categorized as having high agency in this domain using our measure. Moreover, one in ten women (10.3%) who reported making a decision mainly themselves, often assumed to imply high agency, were classified as having low agency using our measure—overall, more than one-quarter of women who reported either joint decision making (27.9%; N = 122) or making the decision about when to have children themselves (25.6%; N = 30) were classified as having low or medium agency using our measure. Similar patterns were also evident in other domains, suggesting that the two measures do in fact differ in important ways.

**Table 5:** Comparison of reproductive decision-making agency with PMA2020-Style question on main decision-maker for three domains of reproductive health, Nepal, 2017

	Measure Based on Performance Monitoring and Accountability (PMA)2020- Style Question: Who Makes Decision?							
	Mainly husband or other		Mainly participant		Joint		Total	
Decision-making agency measure	No.	%	No.	%	No.	%	No.	%
**Domain 1: Agency around when to have children**								
Low agency	164	38.5%	12	10.3%	59	13.5%	235	24.0%
Medium agency	97	22.8%	18	15.4%	63	14.4%	178	18.2%
High agency	165	38.7%	87	74.4%	315	72.1%	567	57.9%
Total	426	100.0%	117	100.0%	437	100.0%	980	100.0%
**Domain 2: Agency around whether to use contraception**								
Low agency	123	37.0%	34	12.1%	30	8.7%	187	19.5%
Medium agency	92	27.7%	47	16.7%	53	15.3%	192	20.0%
High agency	117	35.2%	201	71.3%	263	76.0%	581	60.5%
Total	332	100.0%	282	100.0%	346	100.0%	960	100.0%
**Domain 3: Agency around which method of contraception**								
Low agency	50	14.4%	15	3.5%	5	3.0%	70	7.4%
Medium agency	204	58.6%	92	21.4%	26	15.5%	322	34.0%
High agency	94	27.0%	323	75.1%	137	81.5%	554	58.6%
Total	348	100.0%	430	100.0%	168	100.0%	946	100.0%

Across all three domains, a major area of discordance between the two measures was among women who in the PMA2020-style questions reported that mainly the husband made decisions, usually considered to be the lowest level of agency in decision making. Except in Domain 3, roughly one-third of these women were categorized in the highest agency group for our measure (Domain 1: n = 165 [38.7%]; Domain 2: n = 117 [35.2%]; Domain 3: n = 94 [27.0%]). In Domain 2 (whether to use contraception), 13.5% of women (n = 59) reported joint final decision making using the PMA2020-based measure but were categorized as having low agency. In Domains 1 and 2, 47.4% (n = 87) and 71.3% (n = 201) of women who reported that mainly they themselves were the decision-maker using the PMA202-based measure were in the medium agency category. Finally, for Domain 3, 58.6% (n = 204) of women reporting that the husband mainly makes decisions related to which method of contraception using the PMA2020-based measure were in the medium agency category.

Across all three domains, roughly three-fourths of women who reported they alone were mainly the decision-makers were in the high agency category (Domain 1: n = 87 [74.4%]; Domain 2: n = 201 [71.3%]; Domain 3: n = 323 [75.1%])

## Discussion

In our sample of Nepalese women, we found relatively high levels of agency across our three primary reproductive domains of inquiry: female participants generally felt like they shared their opinion, that their opinions were valued, and that they were ultimately satisfied with the process, in many cases regardless of who made the final decision in each domain. It is possible that these high levels of agency were due to our sampling approach. However, in contexts like Nepal where women are expected to accommodate a husband's or family's expectations, especially around childbearing (Basnyat & Dutta, 2012), the high agency may reflect her satisfaction at fulfilling that expectation, even if it was not something she personally desired.

### Internal consistency and validity of our reproductive decision-making agency variables

Our reproductive decision-making measures were found to be internally consistent within and across domains and with key demographic and reproductive health measures. In areas where we expected women to exercise higher levels of decision making in the reproductive sphere—such as at higher levels of education, employment, in geographic areas with higher socioeconomic status, and in the middle of the parity spectrum—we saw higher levels of agency.

Furthermore, the revised measures proved to be strongly related to several reproductive outcomes, particularly those we felt were theoretically closest to agency and empowerment, even when controlling for a range of factors related to both agency and the outcomes themselves. The close relationship between agency and feelings of reproductive control suggests that the measure is effective in capturing key components of agency that we argue reflect the essence of empowerment.

Interestingly, the measures were less predictive of met need for contraception, which is puzzling and suggests a need for further validation and testing in different contexts and populations. The lack of association may be due to a range of reasons, including the relatively low levels of unmet need for and high levels of use of contraceptives in our sample, or the salience of factors other than agency that shape contraceptive use in this context and for this population.

### Comparison with the PMA2020-style questions

Our main objective of the comparison analyses with the PMA2020-style questions was to understand the advantages our measures have over those standardly administered on quantitative surveys in the field. Concordance between the two approaches was strongest when discussing when to have children and whether to use family planning; both measures generally categorized those making joint decisions as having high agency. In contrast, women were more likely to report being the sole decision-maker (and be classified as having high agency using our approach) when selecting which contraceptive method to use.

However, there were areas of disagreement between our decision-making questions and the PMA2020-style questions that merit further discussion. First, across all three domains, a sizeable proportion of women who reported joint decision making in the PMA2020-style questions were categorized as having low agency in our measure. This discordance was due primarily to two factors. First, many of these women reported in our set of questions that the husband made the decision even when reporting a joint process for the PMA2020-style questions. This may be due to inconsistent reporting among participants or differences in how women respond to single questions about a decision versus a broader set that allow for a more nuanced response. Secondly, many of these women reported wishing they had more influence in the process, suggesting a relative lack of empowerment that is not captured in the PMA2020-syle questions. There are several potential explanations for this discrepancy. This may be due to the different framing of the questions (with the PMA2020-style questions asking about decision making in general while our measures focused on the last time the issue was discussed) and may lead participants to conceptualize the decision-making process in different ways. Both approaches have significant potential limitations—for instance, focusing on specific points in the past, as our approach does, likely introduces elements of recall bias and post-hoc rationalization in reporting that may bias the results^3^. On the other hand, the PMA2020 approach relies on a more hypothetical line of questioning that is less anchored in a specific event and may lead to overreporting of negative or positive experiences or reporting of the ideal rather than the more typical experience.

Our analyses suggest that our approach has some significant advantages over other approaches to exploring reproductive decision-making agency. Our approach allows for the role of voice and power to be explicitly included in the measures of agency in decision making rather than assuming what choice looks like for agency, as is often required with other measures. This allows for greater nuance in measurement and for variation across different reproductive domains. It also makes the approach less reliant on researchers’ decisions about how to prioritize joint or sole decision making in terms of agency. In our measure, for example, women classified as having high agency include both those who made the decision alone and those who made it jointly, depending on their level of engagement in and satisfaction with the decision-making process. In addition, the strong theoretical bases for the domains and measures suggest that they should have a strong potential for broad applicability across other cultures and contexts, even to those that have vastly different power structures and relationship dynamics (e.g., informal, polygamous, or age-disparate unions). Furthermore, this framework should be equally applicable to women and men, including those at different life course stages, although the importance of a domain may vary depending on the population to which the measures are being applied. Finally, our measures are relatively parsimonious, which is an important consideration in large-scale surveys such as the DHS or PMA2020.

### Limitations

There are additional considerations to be made related to our measures. First, these findings suggest further research is needed, both to establish the reliability of our measures in other contexts and to assess how they compare to existing measures. For example, test-retest reliability can help further established external reliability, particularly in settings where intra-household and intra-couple dynamics are different than in Nepal.

It is clear however, that efforts to more comprehensively assess feelings of satisfaction with the decision-making process and incorporate those into measures of decision making such as those used by PMA2020 should be explored. This will ensure that these measures more closely match the theoretical foundations for empowerment and agency and improve the ability of these measures to effectively predict feelings of reproductive control. It may also prove useful in disentangling the ways in which sole and joint decision making are related to agency, thus addressing an important debate in the field. Additional research with couples and men may also shed light on this dynamic.

To be transferable to other contexts, formative research should inform decisions around the wording of specific questions, which may need to be modified to reflect cultural nuances. In addition, these questions are reliant on a couple having discussed an issue; in contexts where verbal communication is minimal or for some issues for which there is less verbal discussion (e.g., having sexual intercourse), these questions may not work well. Relatedly, these measures only capture one component of empowerment—decision-making agency—and should be further assessed and understood as related to other elements of empowerment, such as critical consciousness, and other external factors, such as cultural and gender norms, whenever possible.

Finally, the findings from this study provide further support for the need to consider a broader range of reproductive domains in measurement and to reconsider the types of outcomes that we can expect to be strongly related to agency. Researchers must consider the ways in which agency varies by type of reproductive decision being made and the suitability of focusing solely on outcomes such as use of contraception. We argue that the ability of individuals to exert control over their reproductive lives is a more appropriate outcome to focus on and should be included in more surveys.

## Conclusion

These analyses suggest that our measures of agency in reproductive decision making may provide additional information that current measures do not, allowing for a more accurate measurement of agency that may help address some of the challenges the field has faced in understanding how agency in reproductive decisions influences behavioral outcomes. These analyses suggest that our measures are strongly related with feelings of reproductive control and can be effective in predicting the ability of women to exert control over their reproductive lives. However, further replication in different social contexts is required to fully understand how effective these measures are more broadly and what value they add, if any, to existing approaches.

## Ethical statement

All authors hereby declare that we understand the Ethical Guidelines for Journal Publications and that we have, to the best of our knowledge, adhered to ethical standards for this research and manuscript. This goes for all categories mentioned: reporting standards, data access and retention, originality and acknowledgement of sources, multiple redundant or concurrent publication, confidentiality, authorship of paper, declaring of competing interests, notification of fundamental errors, image integrity and clinical trial transparency.

## Figures and Tables

**Table:** 

Question	Response options
1. When discussing *[insert topic from base question],* I want to know if you shared your opinion about what you wanted with your husband. Would you say that you *(read aloud options to participant):*	Shared your opinion
	Wanted to share your opinion but did not feel comfortable so did not share
	Wanted to share your opinion but did not think opinion would be valued so did not share
	Had the same opinion as husband
	Did not share your opinion because the issue did not matter to you
	Don't recall/don't know
	Participant refused to answer
2. Do you think your opinion was valued?	Was valued
	Was not valued
	Don't recall/don't know
	Participant refused to answer
3. Who had the final say on *[insert topic from base question]*?	Myself
	Husband
	Myself and husband
	Mother-in-law
	Other (specify______)
	No decision made
	Participant refused to answer

4. Would you prefer to have had more influence in the decision about *[insert topic from base question]* less influence, or were you happy with your level of influence?	More influence
	Less influence
	Satisfied
	Unsure
	Participant refused to answer

## Footnotes

1 In addition, we conducted 200 exploratory quantitative surveys with men, using the same tools. Their results are not included in this analysis except as a comparison to the women's data for internal consistency checks.

2 DHS definition includes currently pregnant women's level of wantedness for their pregnancy (wanted the pregnancy at that time; wanted the pregnancy later; did not want the pregnancy) in addition to the disconnect between reproductive desires and use of contraception by nonpregnant women.

3 We conducted several tests to assess the sensitivity of the results to the length of the recall period (i.e., time since the last discussion about the topic) and found no systematic evidence that this influenced the findings.
